# Impact of Be EPIC‐VR training on person‐centered communication in dementia care

**DOI:** 10.1002/alz70858_103266

**Published:** 2025-12-26

**Authors:** Marie Y. Savundranayagam, Annette Schumann, George Philip, Grace Malheiro, Jennifer L. Campos, Joseph B Orange

**Affiliations:** ^1^ Western University, London, ON, Canada; ^2^ KITE ‐ Toronto Rehabilitation Institute, University Health Network, Toronto, ON, Canada

## Abstract

**Background:**

Be EPIC‐VR is a novel person‐centered communication training program for frontline healthcare workers who support persons living with dementia. The aim of this study was to investigate the effectiveness of Be EPIC‐VR on increasing frontline staff's use of person‐centered communication (PCC) during care interactions.

**Method:**

Personal support workers were assigned to either the immediate training group (*N* = 26) or the wait‐list control group (*N* = 18). Participants engaged in a 10‐minute video‐recorded interaction with a virtual avatar depicting a person living with dementia pre‐ and post‐training. Conversation data was transcribed into communication units, coded if indicated and analyzed by frequency of occurrence for the use of PCC. Outcome measures were a) the proportions of Be EPIC‐VR's PCC units (coded as recognition, negotiation, facilitation, validation) and b) the proportions of missed opportunities for PCC (coded as omissions or alternatives).

**Result:**

A 2 (Group: immediate training vs. control) by 2 (Time: pre vs. post) mixed ANOVA on the proportion of PCC units revealed a significant group by time interaction (*F*(1, 42) = 12.86, *p* < .001, η_p_^2^ = .24). Simple effects analyses showed that the immediate training group exhibited a significant increase in PCC from pre‐ to post‐training, whereas there was no change for the control group. Similarly, the ANOVA for missed opportunity revealed a significant group by time interaction (*F*(1, 42) = 17.28, *p* < .001, η_p_^2^ = .29). Follow‐up simple analyses showed that the immediate training group exhibited a significant decrease in missed opportunities for PCC from pre‐ to post‐training, whereas the control group had no change. Additional one‐way repeated measures ANOVA analysis combined participants who completed Be EPIC‐VR (immediate training and waitlist group once they completed training). There were significant increases in PCC (*F*(1,43)=34.19, *p* < .001, η_p_^2^=.44) and significant decreases in missed opportunities (*F*(1,43)=34.59, *p* < .001, η_p_^2^=.45) from pre‐ to post‐Be EPIC‐VR training in this combined group.

**Conclusion:**

Findings show that Be EPIC‐VR enhanced person‐centered communication, which is essential for optimizing quality of care. The findings highlight the importance of performance‐based outcomes when assessing person‐centered care interventions.

